# Vieussens' Arterial Ring and Exercise-Induced Polymorphic VT: Approach to Diagnosis and Management

**DOI:** 10.1016/j.jaccas.2025.104674

**Published:** 2025-08-20

**Authors:** Ali H. Jafry, Aamer Ubaid, Amit Thosani, Joshua Silverstein, Georgios Lygouris, Indu Poornima

**Affiliations:** AHN Cardiovascular Institute, Allegheny General Hospital, Pittsburgh, Pennsylvania, USA

**Keywords:** coronary anomaly, exercise, sudden cardiac death, ventricular tachycardia, Vieussens' ring

## Abstract

**Background:**

Vieussens' arterial ring (VAR) is an embryologic remnant of the conotruncal circle and a rare coronary anomaly.

**Case Summary:**

A 36-year-old man presented for a stress test for evaluation of exercise-induced palpitations. During the test, he developed sustained polymorphic ventricular tachycardia (VT) that self-terminated. Coronary angiography revealed nonobstructive coronary artery disease and type 3 VAR, with the right coronary artery supplying the entire left system. Extensive testing for other etiologies was negative. A repeat stress test after initiation of beta-blocker showed normal myocardial perfusion and no VT. After multidisciplinary patient-centered discussion, surgical revascularization was not pursued. An implantable cardioverter-defibrillator was inserted for secondary prevention, with no recurrence of VT on follow-up.

**Discussion:**

We report an exceedingly rare case of type 3 VAR and polymorphic VT in a young, otherwise healthy man, with successful conservative management. Multimodality testing and a patient-centered approach are essential for accurate diagnosis and effective management.

**Take-Home Message:**

Conservative management for VAR is reasonable in the absence of demonstrable myocardial ischemia and/or poor surgical candidacy.

## Graphical Abstract

**Figure:**
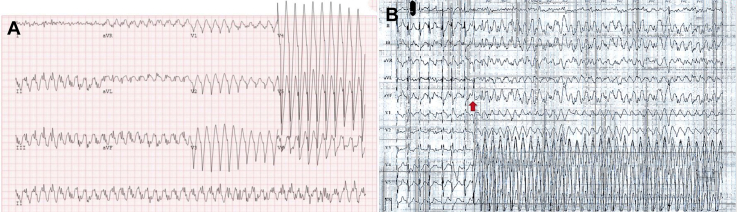

## History of Presentation

**Figure 1:**
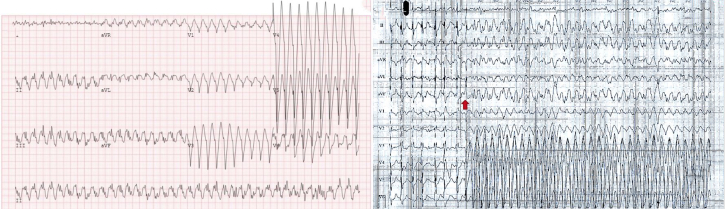
Polymorphic Ventricular Tachycardia Polymorphic ventricular tachycardia initiated by a premature ventricular contraction beginning at 13 minutes and 9 seconds into exercise.

A 36-year-old male presented for an outpatient treadmill electrocardiogram (ECG) stress test for evaluation of palpitations and dizziness that were exacerbated by exercise. Vital signs showed him to be afebrile (36.8 °C), with a heart rate of 64 beats/min, blood pressure of 128/80 mm Hg, respiratory rate of 16 breaths/min, and oxygen saturation level of 99% on room air. The physical examination was entirely normal. The patient displayed above-average functional capacity, achieving 17 metabolic equivalents, but the stress test was complicated by premature ventricular complex–triggered, sustained polymorphic ventricular tachycardia (VT) beginning at 13 minutes and 9 seconds into the test and lasting for 43 seconds before self-terminating (Figures 1A and 1B). No ischemic changes were appreciated on ECG throughout the test. The patient denied chest pain or other symptoms during the episode, however, the study was terminated and the patient was sent to the emergency department for admission and further evaluation.

**Take-Home Messages**

• Type 3 Vieussens' arterial ring may rarely cause sudden cardiac death, possibly owing to intermittent short-lived myocardial ischemia; multimodality testing is critical to reach the correct diagnosis.
• Conservative management is reasonable in the absence of demonstrable myocardial ischemia and/or poor surgical candidacy.

## Past Medical History

The patient had no significant past medical history apart from COVID-19 infection in late 2023. Family history was unremarkable for premature coronary artery disease or sudden cardiac death. He denied smoking, excessive alcohol use, or recreational drug use. He was employed as an elevator mechanic, which required him to perform arc-welding. The patient was initially seen in the emergency department for intermittent palpitations and dizziness that worsened after exertion. Workup, including orthostatic vitals, complete blood count, comprehensive metabolic panel, D-dimer, thyroid-stimulating hormone, ECG, and chest x-ray, was unremarkable, and he was discharged with a 2-week ambulatory monitor and instructed to establish care in the cardiology clinic. At his clinic visit 10 days later, the patient reported excellent functional capacity and was exercising regularly, although his smartwatch had alerted him to intermittent tachycardia, with heart rates up to 160 beats/min. One month after his clinic visit, he presented for the stress test.

## Differential Diagnosis

The initial differential diagnoses for the etiology of VT included catecholaminergic polymorphic VT, anomalous coronary artery with malignant course, hypertrophic cardiomyopathy, arrhythmogenic right ventricular cardiomyopathy, myocarditis, sarcoidosis, lamin A/C cardiomyopathy, arrhythmic mitral valve prolapse/disjunction, and premature coronary artery disease.

## Investigations

**Figure 2:**
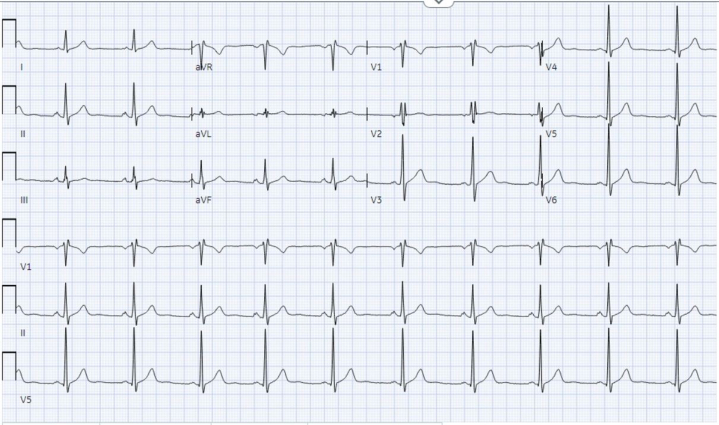
Baseline Electrocardiogram Postexercise electrocardiogram demonstrating normal sinus rhythm, normal axis, intraventricular conduction delay, normal QT interval, and no pre-excitation or repolarization abnormalities.

**Figure 3:**
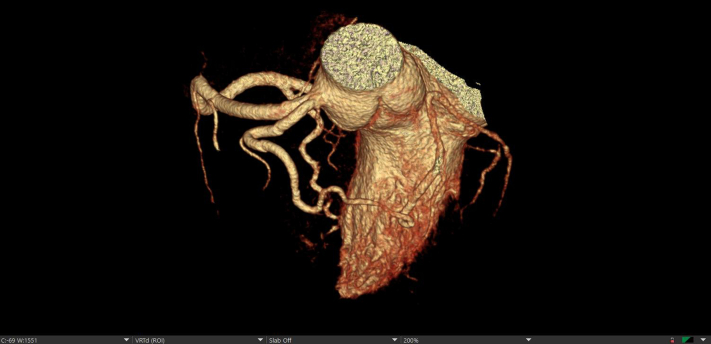
Coronary Computed Tomography Angiography Coronary computed tomography angiography suggestive of a type 3 Vieussens' arterial ring: a coronary anomaly with absent left main and superdominant RCA supplying both LAD and LCx branches. A non-obstructive plaque was noted in the small-caliber mid-distal LAD. LAD = left anterior descending; LCx = left circumflex; RCA = right coronary artery.

**Figure 4:**
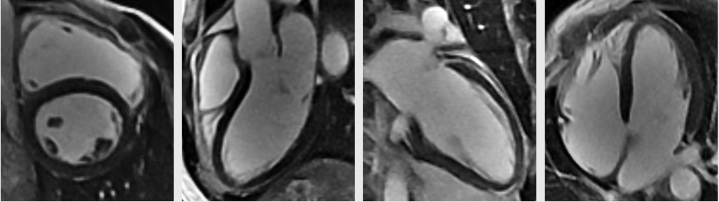
Cardiac Magnetic Resonance Imaging Absence of late gadolinium enhancement on cardiac magnetic resonance imaging.

High-sensitivity troponin T level was normal at 11 ng/L (normal range: 0-22 ng/L); additional laboratory test results were again unremarkable. Baseline ECG showed normal sinus rhythm, normal axis, intraventricular conduction delay, normal QT interval, and no pre-excitation or repolarization abnormalities (Figure 2). The patient's ambulatory monitor results showed paroxysmal supraventricular tachycardia episodes and 2% premature atrial complex burden; interestingly, no VT was noted. Transthoracic echocardiogram on the day of admission showed mild biventricular dysfunction, with left ventricular ejection fraction of 45% to 49% and no significant valvular heart disease (Video 1). Coronary computed tomography angiography (CCTA) showed type 3 Vieussens' arterial ring (VAR), a rare anomalous coronary variant with absent left main and superdominant right coronary artery supplying both left anterior descending (LAD) and left circumflex (LCx) branches (Figure 3). Invasive coronary angiography (ICA) confirmed presence of VAR, demonstrating the conus artery filling the mid LAD with retrograde flow into the proximal LAD, diagonal and LCx; the right posterior ventricular branch was also noted to supply the distal LCx coronary artery, effectively forming a ring at the base of the heart (Videos 2A and 2B). The mid-distal LAD was appreciated as a small-caliber vessel with mild diffuse disease. CCTA and ICA ruled out a malignant coronary course, ostial stenosis/slit-like origin, dynamic compression, and premature coronary artery disease as etiologies. Cardiac magnetic resonance imaging on the third day of hospitalization showed normalization of biventricular function and absence of late gadolinium enhancement, ruling out hypertrophic cardiomyopathy, sarcoidosis, myocarditis, and arrhythmogenic right ventricular cardiomyopathy (Figure 4). A comprehensive genetic panel to assess for arrhythmias and cardiomyopathies was negative, including for catecholaminergic polymorphic VT and lamin A/C cardiomyopathy.

## Management

Low-dose metoprolol tartrate was initiated but could not be uptitrated owing to bradycardia. In the absence of chest pain, troponin leak, or ischemic ECG changes, it was debated whether the VT was truly secondary to myocardial ischemia; after ruling out other etiologies as detailed earlier, adrenergic VT was also discussed as a differential diagnosis. An exercise stress test was decided upon, this time with myocardial perfusion imaging to objectively assess for evidence of ischemia. The patient had no recurrence of VT at peak exercise, again demonstrating above-average functional capacity and actually exceeding exercise time (13 minutes, 57 seconds) and peak heart rate compared with his prior stress test; importantly, myocardial perfusion was normal. A patient-centered multidisciplinary discussion followed, involving the general and interventional cardiology, electrophysiology, and cardiothoracic surgery teams. In the presence of robust left-sided coronary collaterals from the VAR, absence of objective evidence of regional ischemia at peak exercise, and with a small-caliber mid-distal LAD which was a poor target for surgical bypass, it was eventually decided not to pursue immediate surgical revascularization.

**Figure 5:**
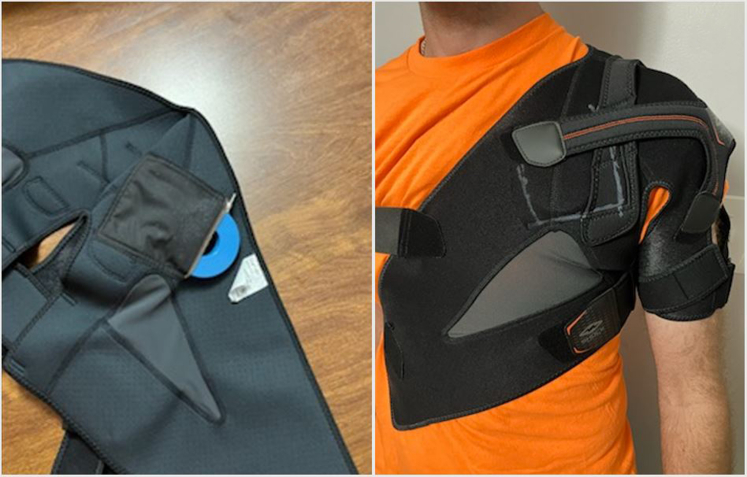
Vest With Magnet Specially designed vest housing a magnet; to be worn by patient during arc-welding.

**Figure 6:**
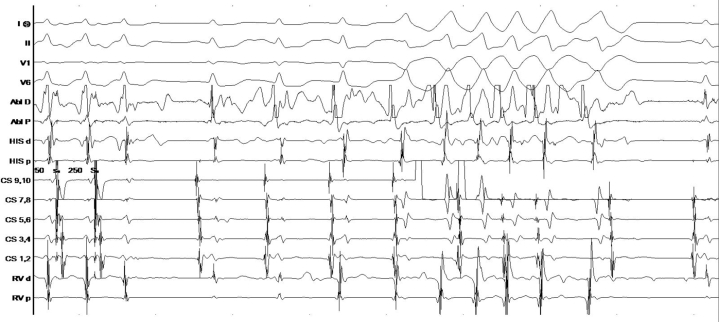
Electrophysiology Study Polymorphic ventricular tachycardia with isoproterenol infusion and burst atrial pacing postablation.

For secondary prevention of sudden cardiac death, as well as his baseline bradycardia limiting beta-blocker uptitration, insertion of a dual chamber implantable cardioverter-defibrillator (ICD) rather than a subcutaneous device was discussed. The patient was unwilling to forego arc-welding, as he relied on it for his livelihood; thus, he initially wore a wearable defibrillator after discharge. A specially designed vest housing a magnet was designed, and he was thereafter agreeable to an ICD (Figures 5A and 5B). The electrophysiology study demonstrated dual atrioventricular nodal physiology and atrioventricular nodal re-entrant tachycardia as the cause of the supraventricular tachycardia, for which a successful slow pathway modification was performed. This was followed by dual-chamber ICD placement for secondary prevention; of note, nonsustained runs of polymorphic VT were noted, with peak isoproterenol infusion and burst atrial pacing (Figure 6). He was extensively counseled to wear the magnet vest only during exposure to arc-welding and to restrict himself to moderate exertion at most.

## Outcome and Follow-Up

The patient was doing well at his postprocedure visit 1 week later; a remote device check 2 weeks later did not reveal any VT or shocks. Metoprolol was switched to atenolol owing to reported fatigue. At his subsequent clinic visit 1 year later, he denied any symptoms with moderate exertion, and device interrogation revealed no episodes of VT.

## Discussion

VAR is a rare epicardial coronary anomaly, first described by and named after the famed French anatomist Raymond de Vieussens in his treatise, “Nouvelles Découvertes sur le Coeur” (New Discoveries on the Heart), published in 1706.^1^ Various observational studies have reported an estimated prevalence of up to 3% in select populations, though prevalence in the general population is likely much lower.^2^ The variant is theorized to be a remnant of the embryonic conotruncal circle rather than a product of neoangiogenesis.^3^ It commonly exists as a connection between the conus artery and the LAD, and it may often be seen in the context of complex congenital heart disease. Previous studies have classified it into subtypes 1a (VAR without vascular pathology), 1b (VAR with vascular pathology, eg, aneurysm/fistula), 2 (VAR with LAD duplication), and 3 (VAR with single coronary artery anomaly), with type 3 being exceptionally rare. Patients are more likely to be male and may present with angina, particularly if coronary artery disease, VAR fistulae, or aneurysms are present; VAR constitutes an important route for collateral circulation in these patients.^4^ ICA is the gold standard for diagnosis, though CCTA has been increasingly effective at detection and classification.^2^^,^^5^

Studies have shown coronary anomalies to be the second most common cause of sudden cardiac death in young, competitive athletes.^6^ Tomura et al^7^ described cardiac arrest in a 16-year-old man with left main coronary atresia and type 3 VAR. The patient underwent coronary artery bypass grafting after demonstration of myocardial perfusion defects. Our case demonstrates a unique instance of exercise-induced polymorphic VT in a young man with type 3 VAR but normal myocardial scintigraphy. As extensive testing for other etiologies was negative, we theorize that type 3 VAR likely triggered myocardial ischemia and caused VT, exacerbated perhaps in the presence of a small LAD. It remains uncertain whether the ischemia was solely attributable to the anatomical anomaly or was amplified by a functional component—namely, epicardial and microvascular spasm within a single coronary circulation causing brief, imaging-silent ischemia. Additionally, we cannot definitively rule out the possibility of a nonischemic mechanism such as adrenergic and Purkinje-related VT, or channelopathic triggers; indeed, coexistence of such uncommon substrates may be observed in rare congenital coronary patterns.

The 2018 American Heart Association/American College of Cardiology guidelines recommend surgery for patients with evidence of coronary ischemia (Class I recommendation) or ventricular arrhythmias (Class IIa recommendation) directly attributable to an anomalous coronary artery.^8^ Management of patients with ventricular arrhythmias in the absence of demonstrable regional myocardial ischemia or those with poor surgical targets, as in our case, is uncertain. Studies have suggested mandatory demonstration of a causal link between coronary anomalies and myocardial ischemia before surgical intervention, especially since ischemia may persist even after surgical correction.^9^ Successful conservative management with exercise restriction and medical therapy has been reported in such cases, including for patients with syncope and abnormal stress tests.^10^

## Conclusions

Type 3 VAR is a unique coronary anomaly that may trigger polymorphic VT due to intermittent myocardial ischemia. A multimodal imaging approach is critical to diagnosis and classification. It is unclear if surgical revascularization provides benefit if myocardial ischemia is not demonstrated; management with medical therapy, exercise restriction, and an ICD is reasonable in such cases.

## Funding Support and Author Disclosures

The authors have reported that they have no relationships relevant to the contents of this paper to disclose.

## Appendix

Video 1 Transthoracic Echocardiogram Transthoracic echocardiogram on the day of admission showed mild biventricular dysfunction, with a left ventricular ejection fraction of 45% to 49%.

Video 2a and 2b Invasive Coronary Angiogram Coronary angiogram confirmed the presence of Vieussens' arterial ring. Here the conus branch is seen filling the mid-distal LAD, with retrograde flow into the proximal LAD, diagonal and LCx branches. The small-caliber mid-distal LAD was noted to have mild, nonobstructive coronary artery disease. LAD = left anterior descending; = .

## References

[1] Vieussens R. (1706).

[2] Doğan N., Dursun A., Özkan H. (2019). Vieussens' arterial ring: a rare coronary variant anatomy. Diagn Interv Radiol.

[3] Klein L.W., Campos E.P. (2019). The embryologic origin of Vieussens' ring. J Invasive Cardiol.

[4] Christodoulou K.C., Stakos D., Androutsopoulou V. (2023). Vieussens' arterial ring: historical background, medical review and novel anatomical classification. Cureus.

[5] Bamoshmoosh M., Fanfani F., Volpe C. (2014). Vieussens' arterial ring visualized by MDCT. Open J Radiol.

[6] Maron B.J., Doerer J.J., Haas T.S., Tierney D.M., Mueller F.O. (2009). Sudden deaths in young competitive athletes: analysis of 1866 deaths in the United States, 1980–2006. Circulation.

[7] Tomura N., Nakagami T., Yamaguchi S., Yaku H., Patel P.A. (2020). Sudden cardiac arrest of a 16-year-old boy with left main coronary artery atresia: a case report. Eur Heart Journal-Case Rep.

[8] Stout K.K., Daniels C.J., Aboulhosn J.A. (2019). 2018 AHA/ACC guideline for the management of adults with congenital heart disease: a report of the American College of Cardiology/American Heart Association task force on clinical practice guidelines. J Am Coll Cardiol.

[9] Brothers J.A., McBride M.G., Seliem M.A. (2007). Evaluation of myocardial ischemia after surgical repair of anomalous aortic origin of a coronary artery in a series of pediatric patients. J Am Coll Cardiol.

[10] Kaku B., Shimizu M., Yoshio H., Mizuno S., Kanaya H., Mabuchi H. (1996). Clinical features and prognosis of Japanese patients with anomalous origin of the coronary artery. Jpn Circ J.

